# Attention problems and language development in preterm low-birth-weight children: Cross-lagged relations from 18 to 36 months

**DOI:** 10.1186/1471-2431-11-59

**Published:** 2011-06-29

**Authors:** Luisa A Ribeiro, Henrik D Zachrisson, Synnve Schjolberg, Heidi Aase, Nina Rohrer-Baumgartner, Per Magnus

**Affiliations:** 1 Division of Mental Health, Norwegian Institute of Public Health, Post Box 4404, Nydalen, Oslo 0403, Norway; 2 Division of Epidemiology, Norwegian Institute of Public Health, Post Box 4404, Nydalen, Oslo 0403, Norway

**Keywords:** MoBa, The Norwegian Mother and Child Cohort Study, premature, low birth weight, attention, language

## Abstract

**Background:**

Research has highlighted a series of persistent deficits in cognitive ability in preterm low-birth-weight children. Language and attention problems are among these deficits, although the nature of the relation between attention and language in early development is not well known. This study represents a preliminary attempt to shed light on the relations between attention problems and language development in preterm low-birth-weight children.

**Methods:**

The aim of this study was to analyse reciprocal influences between language and attention problems from 18 to 36 months. We used maternal reports on attention problems and language ability referring to a sample of 1288 premature low-birth-weight infants, collected as part of the Norwegian Mother and Child Cohort Study (MoBa). A sample of children born full-term was used as the control group (N = 37010). Cross-lagged panel analyses were carried out to study reciprocal influences between attention problems and language.

**Results:**

Language ability at 18 months did not significantly predict attention problems at 36 months, adjusting for attention problems at 18 months. Attention problems at 18 months significantly predicted changes in language ability from 18 to 36 months, pointing to a precursor role of attention in relation to language in children born preterm. Gender, age corrected for prematurity, and mother's education emerged as important covariates.

**Conclusions:**

Preliminary evidence was found for a precursor role of early attention problems in relation to language in prematurity. This finding can contribute to a better understanding of the developmental pathways of attention and language and lead to better management of unfavourable outcomes associated with co-morbid attention and language difficulties.

## Background

Several studies of the developmental outcome of premature low-birth-weight infants have highlighted a series of persistent deficits in cognitive ability across the life span [1-3]. Children born preterm seem to be at increased risk for atypical trajectories of cognitive development and are overrepresented among those with attention problems, language difficulties, and poor school performance [4].

Preterm birth is likely to impact significantly on brain development since the central nervous system of the premature baby is not fully prepared to function independently outside the intra-uterine environment [4]. In fact, neuroimaging research has identified anatomical abnormalities as the result of premature birth, such as smaller hippocampus [5], lower gray-to-white-matter ratio [6], and smaller cerebellum [7]. These structural changes in the brain are thought to be related to deficits in cognitive functioning [8].

Moreover, other neonatal medical complications are also common in prematurity such as septicaemia, intraventricular haemorrhage, chronic lung disease, apnea, bradycardia, and so on [9]. Medical complications and treatment interventions, coupled with prolonged hospital stays, can impact negatively on brain development and contribute to accentuate long-term neurobehavioral deficits [10].

Environmental factors can also play an important role in developmental outcomes of prematurity. For example, preterm infants from high SES families show fewer problems later in life when compared to infants from low SES families [11]. Frontal and posterior brain regions implicated in language and attention skills may be particularly affected by environmental factors [8]. In fact, programmes to decrease environmental risk in preterm infants have been shown to improve neurobehavioral functioning involving frontal and occipital areas [12]. Gender differences have also been observed. Boys seem to attain less favourable cognitive outcomes than girls [13], although this finding is not consistent across studies [14].

Language difficulties are prevalent in premature children and include articulation problems and expressive language delays, which can manifest themselves as poor vocabulary and grammar. Difficulties with phonological awareness are also common and predict later poor reading and writing [15,16]. In fact, preterm birth is likely to have long-term consequences, affecting linguistic development beyond preschool [15].

There is some controversy regarding the conceptualization of language development as either solely dependent on language processes or as tied to more domain-general cognitive processes [17]. Several studies have attributed language impairments in premature infants, especially those born extremely preterm, to a general cognitive deficit affecting several areas of functioning [16]. However, not much is known about the potential impact of more general cognitive mechanisms, such as attention, on language development [17,18].

Studies on executive function and attention skills in premature infants have considered the general-cognitive-deficit hypothesis as having insufficient explanatory power, suggesting that attention deficits might be a specific area of weakness in preterm children [19]. A recent meta-analysis has confirmed that attention skills are especially problematic in preterm children [20]. Attention problems seem to occur even in cases of non-extreme low birth weight [21]. Factors such as gestational age, gender, and environmental factors (e.g., mother's SES) strongly contribute to the extent of attention problems [20].

Attention and language become increasingly interdependent throughout development. Initially, the infant's attention system is basically comprised of orienting processes characterized by visual exploration of the environment. Later on, by the end of the first year, a second attentional system, the *executive attention system*, emerges. It enables the child to engage in goal-oriented activity and inhibition of behaviour. This system is also closely associated with language development by enabling the production of verbal cognitions to control behaviour [22].

Several studies have found high co-morbidity of cognitive deficits early in development in both full- and preterm children [1,23,24]. In particular, there is a frequent co-morbidity of language and attention problems [4,25]. Neurological studies have shown that children with language disorder show differential activation of cerebral regions involved in several attention processes [26].

Children with better attention are expected to be faster in language learning because they tend to be better at following adults' gazes, at engaging in joint attention, and at tracking the referents of other people's verbalizations [17]. Dysregulation of attention and arousal are expected to interfere with the ability to maintain a focused state, affecting children's opportunity to engage in social interactions and decreasing opportunities for language learning.

Not many studies have addressed relations between attention and language in preterm children. According to the general-cognitive-deficit hypothesis, cross-sectional associations between these two skills would be expected both in infancy and early childhood. Moreover, since attention seems to be a particularly weak area for preterm children [19], it is possible that early attention problems have a precursor role in predicting later language ability. In fact, early attention deficits have been identified as important in signalling future cognitive difficulties [4], such as those related to language processing. Language problems in infancy are difficult to diagnose [27] and the identification of early cognitive markers of language impairment can facilitate earlier detection and increase our understanding of underlying mechanisms associated with less favourable outcomes of prematurity.

Despite well-documented co-morbidity between language and attention problems in children born preterm [4], little is known about early pathways relevant to these conditions. The aim of this study is to investigate reciprocal influences of language and attention problems in preterm low-birth-weight (*PLBW*) children at 18 and 36 months. We seek to explore associations between early attention problems and later language ability and associations between early language ability and later attention problems. We hypothesise that attention problems are a precursor of language ability in *PLBW *children, in accordance with studies emphasizing attention as a particularly problematic area in this group. We expect attention problems to predict change in language ability better than language ability predicts change in attention problems. This would support a *precursor role *of attention problems in relation to language ability [28]. Maternal perceptions of children's attention problems and language ability at both 18 and 36 months provide the basis for the present preliminary study. Both concurrent and predictive relations between language and attention problems will be examined in a sample of over a thousand children born preterm and with low birth weight.

## Method

### Participants

Data referring to a sample of 1288 Preterm Low-Birth Weight (*PLBW*) children (700 girls, 588 boys) born before 38 completed weeks of gestation (range 23.9-37.9) and with birth weight below 2500 g (500-2499) were included in the present study. Around 10% of the premature children were born with birth weight below 1500 g (Very Low Birth Weight, n = 132) and 3% below 1000 g (Extreme Low Birth Weight, n = 40). Proportion of type of preterm delivery was as follows: spontaneous (20.2%), medically indicated (33.4%), and multiple births (34.2%). A group of 37010 children born full-term (38 completed weeks or more; range 38.0-46.6) and with normal birth weight (> 2499 g; range 2500-6320) comprised the control group. Around 11% of the *PLBW *children (n = 139) presented with respiratory distress syndrome at birth. Almost no children suffered from intracranial bleeding at birth (< 1%). Children with severe syndromes and neurodevelopmental conditions were excluded from the analyses (e.g., Down Syndrome), as well as those with impaired/reduced hearing or impaired vision. Children with non-Norwegian parents (mother and/or father) were also excluded. Demographic and medical information for *PLBW *children and controls can be found in table 1.

**Table 1:** Medical and demographic characteristics of the PLBW (n = 1288) and the control (n = 37010) groups

	*PLBW*		*Control Group*	
	***M***	***SD***	***M***	***SD***

Birth weight (g)	2002.4	424.6	3705.4	474.2

Gestation (wks)	33.7	2.6	39.9	1.2

Apgar 1 min.	8.0	1.7	8.7	1.1

Apgar 5 min.	9.0	1.1	9.4	0.7

Maternal age	29.6	4.7	30.0	4.4

Gender (% male/female)	46/54		51/49	

Maternal education (% university graduates)	62		66	

### Measures & Procedure

The data used in this study were drawn from the Norwegian Mother and Child Cohort Study (MoBa, http://www.fhi.no/morogbarn). This is a study conducted at the Norwegian Institute of Public Health including a cohort of more than 100 000 pregnant women recruited from 1999 to 2009. Participants were contacted in the sequence of routine ultrasound examinations offered to all women in Norway between the 17^th^and 18^th^week of pregnancy. Participation rate was 38.5%. Data collection includes questionnaires to mothers and fathers, and biological samples from parents and children. The MoBa questionnaires were administered at three time points before birth and also when the child was 6 (T4), 18 (T5), and 36 (T6) months. There are several measures tapping different areas of child development such as language, motor skills, behaviour, eating habits, social skills and so on [29]. Informed consent was obtained from the mothers and the study was approved by The Regional Committee for Medical Research Ethics and the Norwegian Data Inspectorate.

For the purpose of the current study, we used data on language ability and attention problems at T5 (18 months) and T6 (36 months). For the assessment of language ability at 18 and 36 months, items from the Norwegian version of the *Ages and Stages Questionnaires *(ASQ) [30] were used. Attention problems were assessed with items from the *Child Behavior Checklist (CBCL) *[31] and the DSM-IV [32]. Information about children's weight at birth, gestational age, and medical birth status was obtained via the Medical Birth Registry of Norway Data, which has been linked to MoBa. A subsample of 1288 *PLBW *children (gestational age < 38 weeks & birth weight < 2500 g), whose mothers had completed the relevant assessments, was drawn from the larger sample. Children, born full-term (38 completed weeks or more) and with normal birth weight (at least 2500 g) served as the control group (full-term children with low birth weight and premature children with normal birth weight were not included in the present study).

The questionnaires were sent out to the mothers enrolled in the study when their target child completed 18 and 36 months. For the attention items, mothers were requested to assess their children's behaviours based on the last two months (e.g., *To what extent are the following statements true for your child's behaviour during the last 2 months*?). For the language measures, no specific instruction was given since most items were based on present behaviours the mother could test before completing the questionnaires (e.g., *When you ask him/her does your child go to another room to find a familiar toy or object?*).

Due to the logistics involved in such a large-scale longitudinal study, mothers of *PLBW *and control children received the questionnaires at the same time. Therefore, *PLBW *children were not assessed at age corrected for prematurity. The issue of correcting for prematurity is controversial, but the general consensus seems to be the use of corrected age up to 18-24 months [33]. Our outcome variables were assessed after the age of 24 moths and our sample included a very low percentage of very preterm/very low birth weight children (those for whom corrected age is particularly important). However, since one of our measurement times preceded 24 months, we controlled for the effect of age corrected for prematurity including it as a covariate in an extended cross-lagged model.

### Analytic strategy

Statistical analyses were firstly conducted for the group of interest composed of *PLBW *children. In a second step, results from the control group were also analysed. We used a Structural Equation Modelling (SEM) approach to carry out the analyses. All indicators were ordered categorical and therefore the WLSMV (*mean and variance-adjusted weighted least-squares*) estimation procedure was used. This procedure has the advantage of not assuming multivariate normality. Model fit was evaluated by using the Comparative Fit Index (CFI, [34]), the Tucker-Lewis Fit Index (TLI, [35]) and the Root Mean Square Error of Approximation (RMSEA, [36]). TLI and CFI values over .90 indicate good model fit, as well as RMSEA values of .05 or lower [37]. All the SEM analyses were conducted by using the Mplus Software Package (version 6.0) [38].

A cross-lagged panel design was used to test the predictive relation between language ability and attention problems across two time points. The design accounts for time precedence and for multivariate dependences of the predictor variables [39]. The cross-lagged design comprises: a) correlation between attention and language at 18 months; b) paths from 18 to 36 months attention, and from 18 to 36 months language, representing the stability of each construct over time, adjusted for the cross-lagged path of the other construct (e.g., the path from 18- to 36-month language is adjusted for 18-months attention); c) cross-lagged paths from attention at 18 to language at 36 months and language at 18 to attention at 36 months (adjusted for stability within each construct), representing the influence of e.g. attention at 18 months on *change *in language between 18 and 36 months (i.e. residual change); d) correlation between the residuals of attention and language at 36 months (i.e. change from 18 months). Note that cross-lagged models provide tests of reciprocal influences between constructs over time, not of causality [40].

## Results

### Data preparation and descriptive statistics

Four latent variables were constructed to assess language ability and attention problems at 18 and 36 months. The latent variables measuring language ability at 18 (L18) and 36 (L36) months were created by grouping indicators corresponding to ASQ items (communication scale). The latent variables measuring attention problems at 18 (At18) and 36 (At36) months resulted from grouping CBCL items reflecting attention problems, as well as three additional items included in the DSM-IV inattention scale. For a complete list of all indicators included in the four latent variables, see Additional File 1: Appendix 1. All indicators were categorical and with three response categories (*1.yes/true, 2.sometimes/sometimes true, 3.no/not true*). Response frequencies for each questionnaire item included in the study can be seen in table 2. Frequency of missing values at T5 was around 5%; attrition at T6 was around 38%. For the analyses using covariates, missing data on covariates was imputed (Expectation-Maximization) in the Statistical Software Package, SPSS 17.0.

**Table 2:** Frequency of responses to the latent variables language ability and attention problems

	*PLBW* *Frequencies % (1/2/3) *±	*Control Group* *Frequencies % (1/2/3) *±
Language 18 months: item 1	67.2/23.8/9.0	84.5/13.3/2.2

item 2	45.8/7.4/46.8	67.9/7.3/24.8

item 3	47.8/32.8/19.4	65.9/25.8/8.3

Attention 18 months: item 1	62.5/33.5/4.0	64.4/31.9/3.7

item 2	14.0/60.0/26.0	13.1/63.4/23.5

item 3	67.2/28.3/4.5	67.9/28.8/3.3

Language 36 months: item1	98.5/1.2/0.3	99.0/0.9/0.1

item 2	98.2/1.0/0.8	98.7/1.1/0.2

item 3	97.0/1.7/1.3	97.9/1.4/0.7

item 4	91.5/8.3/0.2	94.8/4.9/0.3

item 5	91.2/7.6/1.2	94.3/5.0/0.7

item 6	48.8/36.8/14.4	61.1/30.2/8.7

Attention 36 months: item 1	61.7/34.3/4.0	66.4/31.2/2.4

item 2	29.3/55.7/15.0	30.9/55.6/13.5

item 3	18.6/67.5/13.9	15.7/69.0/15.3

item 4	63.3/34.2/2.5	66.9/31.4/1.7

item 5	56.8/40.0/3.2	60.7/36.8/2.5

± Language ability: 1/2/3 = *yes/sometimes/not yet*; Attention problems: 1/2/3 = *not true/sometimes true/very true*. Category 1 represents better language skills and less attention problems, and category 3 worse language skills and more attention problems.

### Cross-lagged models

We followed a two-step sequence typical when analysing hybrid models. Firstly, we determined the fit of the measurement model for the *PLBW *children by conducting confirmatory factor analyses (CFA) based on the latent variables language ability and attention problems.

At 18 months, a two-factor solution was used including the latent variables L18 (language) and At18 (attention problems). One loading for each latent factor was fixed to one to set its scale. A good model fit was obtained (CFI = .99, TLI = .99, RMSEA = .03). Standardized factor loadings for the latent variable L18 ranged from .51 to .94 and for the latent variable AT18 ranged from .62 to .74. All factor loadings were statistically significant at the .05 level. There was a small but significant correlation between the latent variables L18 and At18 (r = .14, p < .001).

For the time point 36 months, a two-factor solution was also used including the latent variables L36 (language) and At36 (attention problems). The model attained a good fit (CFI = .98, TLI = .98, RMSEA = .04). Standardized factor loadings for the latent variable L36 ranged from .63 to .80 and for the latent variable AT36 ranged from .39 to .88. All factor loadings were statistically significant at the .05 level. There was a modest significant correlation between L36 and At36 (r = .28, p < .001).

The latent variables attention and language had some overlapping indicators at 18 and 36 months. Therefore, in order to test measurement invariance over time, equality constraints were imposed for corresponding factor loadings at T5 (18 months) and at T6 (36 months). The model provided similar parameter estimates and identical overall model fit (CFI = .97, TLI = .96, RMSEA = .03) to the unconstrained CFA model (where the factor loadings were allowed to vary freely), suggesting that the factor loadings were invariant between the two occasions.

Next, we tested the structural model by evaluating cross-sectional and cross-lagged panel associations. Errors from identical indicators were allowed to correlate between the two time points. A good model fit was attained (CFI = .97, TLI = .96, RMSEA = .03). The model showed significant correlations between the latent variables language ability and attention problems at 18 months (r = .15, p < .001) and between the residuals at 36 months (r = .29, p < .001). Both language ability and attention problems were quite stable over time, adjusted for reciprocal influence (language: .67; attention: .56). The lagged path from At18 to L36 was significant at the .05 level (β = .15), meaning that attention problems at 18 months predict level of change in language ability from 18 to 36 months. The lagged path from L18 to AT36 was non-significant (β = .10, p > .05).

The same set of procedures was followed to test the fit of the measurement model for the control group. Similar fit indices and parameters were found. The structural model was finally tested in a separate cross-lagged analysis. The model attained a good fit (CFI = .98, TLI = .98, RMSEA = .02). Significant cross-sectional associations between language ability and attention problems were found at 18 (r = .16) and 36 months (r = .24), as well as stability of these two variables over time (language: .64, attention: .57). Both lagged paths from L18 to At36 (β = .09) and from At18 to L36 (β = .06) were statistically significant at the .05 level.

In order to assess whether the parameter estimates of the cross-lagged effects were comparable between the groups (*PLBW *and controls), we computed a base-line model with no equality constraints between parameters of the two groups. Next, the factor loadings were constrained to be equal for both groups and a DIFFTEST was conducted between the two models (the DIFFTEST is used to obtain an accurate chi-square difference test when the WLSMV estimator is used). No measurement invariance was found between the groups, that is, the meaning of the latent constructs differed between *PLBW *and control children. For this reason, the groups were analysed in separate models.

### PLBW model with covariates

**Figure 1:**
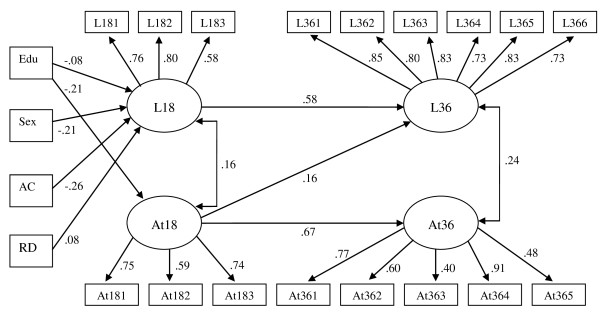
***PLBW *cross-lagged model with covariates for language ability and attention problems at 18 and 36 months: Edu = mother's education, Sex = child's gender, AC = age corrected for prematurity (calculated by subtracting number of days premature from chronological age), and RD = respiratory distress syndrome at birth**. Non-significant paths are omitted for simplicity.

The baseline model was extended to include important variables associated with prematurity and which could potentially influence model parameters: age corrected for prematurity and respiratory distress syndrome at birth (present in 11% of the *PLBW *children). Child's gender and mother's education were also entered into the cross-lagged model as covariates. The extended cross-lagged model with covariates attained a good fit (CFI = .96, TLI = .95, RMSEA = .03) and the parameters were similar to those of the unadjusted model. The lagged path from At18 to L36 was significant (β = .16, p < .05). The lagged path from L18 to AT36 was non-significant (β = .09, p > .05). Gender and age corrected for prematurity emerged as the best predictors of language ability at 18 months (L18) and mother's education as the best predictor of attention problems at the same age (At18) (see Figure 1).

### Control-group model with covariates

**Figure 2:**
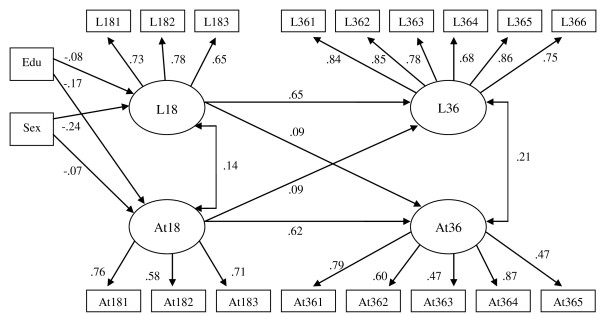
**Control group cross-lagged model with covariates for language ability and attention problems at 18 and 36 months: Edu = mother's education, Sex = child's gender**.

A model with covariates was also tested for the control group. Child's gender and mother's education were added as covariates. Parameters were similar to the unadjusted model and a good model fit was attained (CFI = .98, TLI = .97, RMSEA = .02). Both cross lagged paths were statistically significant. Gender remained the best predictor of language ability at 18 months (L18) and mother's education the best predictor of attention problems at the same age (At18) (see Figure 2)

### Preliminary subgroup analyses

The number of very premature/very low birth weight children in this sample was relatively low. Nevertheless, a preliminary model with covariates was constructed including a subgroup of 218 premature children born before 33 weeks of gestation and weighing less than 1700 g (there were not enough children with birth weight below 1500 g to run the model). For this subgroup, the first three indicators of L36 had to be dropped since they produced categories with too few observations due to reduced sample size. Results were similar to those found for the overall group of *PLBW *children. The cross-lagged path from At18 to L36 approached significance (β = .31, p = .07). The path from L18 to At36 was non-significant (β = .02, p = .90). The overall model attained a good fit (CFI = .92, TLI = .90, RMSEA = .04). Moreover, similar results were found for the covariates although gender, mother's education, and presence/absence of respiratory distress syndrome did not attain significance as predictors of language at 18 months. Only age corrected for prematurity (β = -.36) attained significance as a predictor at the .05 level. Finally, besides mother's education (β = -.35), gender (β = -.23) was also a significant predictor of attention problems at 18 months in this subgroup of very preterm children, with boys performing worse than girls.

## Discussion

In the current study, we investigated reciprocal influences of language and attention in premature low-birth-weight children. Our results lend support to the hypothesis of a precursor role of early attention problems in relation to language in *PLBW *children, in accordance with studies emphasizing attention as a particularly problematic area in this group [19].

Initial *PLBW*-group analyses revealed that attention problems and language ability were quite stable over time when adjusted for reciprocal influence. Moreover, significant albeit modest cross-sectional associations between attention problems and language were observed both at 18 and 36 months, in line with previous studies [22]. Given that attentional and linguistic processes become increasingly interdependent throughout development [22], it is perhaps not surprising that associations between these two skills were modest, especially at 18 months. We found slightly stronger associations between residual change in language ability and attention problems at 36 months, both for *PLBW *and control children, possibly reflecting a trend toward higher interdependence between the two variables over time. Moreover, as anticipated, attention at 18 months emerged as a significant predictor of language ability at 36 months (adjusting for language ability at 18 months).

Similar relations between parameters reflecting stability in attention problems and language ability were observed in *PLBW *children and controls, as well as similar patterns of cross-sectional associations. However, the two models differed with regards to lagged paths. For the *PLBW *children, the path from attention problems at 18 months to language ability at 36 months was larger than that observed for the control group. This could potentially suggest a stronger predictive role of early attention problems on later language in *PLBW *children than in controls. However, since the latent variables seem to have a different meaning in the two groups (lack of group measurement invariance), one cannot determine whether the difference between lagged coefficients is statistically significant. That is, one cannot determine whether level of attention problems at 18 months provides more information about (adjusted) language ability at 36 months in *PLBW *children than in controls.

The within-group relation between the two lagged paths was also distinct between the groups. In the control group, both paths (from attention at 18 months to language at 36 months and from language at 18 months to attention at 36 months) were statistically significant, reflecting equivalent reciprocal influences between attention and language (we tested also the model in a randomly selected subsample of approximately the same size as the *PLBW *group and both cross-lagged paths remained significant). In the *PLBW *group, the path from attention problems at 18 months to language ability at 36 months was larger (and statistically significant) than the (non-significant) path from language ability at 18 months to attention problems at 36 months. It seems therefore that the general-deficit-hypothesis [16] might lack some explanatory power in *PLBW *children. Attention problems might deserve special consideration in this group, instead of being regarded as another manifestation of an underlying general cognitive deficit [19].

Associations between attention problems and language ability, both cross-sectional and cross-lagged, were somewhat low when compared to associations between the same constructs over time. The most robust finding of the study is that early attention problems are the best predictors of later attention problems and that early language ability is the best predictor of later language ability. However, when it comes to reciprocal influences (which are the main focus of this paper) it is noteworthy that, in *PLBW *children, the latent variable attention problems at 18 months was as good a predictor of adjusted language ability at 36 months as it was of contemporary language ability (18 months). This finding seemed to be unique to the *PLBW *group and points to modest but non-negligible evidence supporting a precursor role of attention problems in prematurity.

With regards to the *PLBW *model adjusted for covariates, age corrected for prematurity was an important predictor of language ability at 18 months. Even within this group of children born before 38 weeks of gestation, lower gestational age (reflected in younger age corrected for prematurity) predicted poorer language skills over and above the effect of other covariates such as gender and mother's education. In fact, gestational age has been considered as a better indicator of developmental and neurological maturity than birth weight [41]. Gender was an equally important predictor of language ability at 18 months. Premature boys showed significantly poorer language ability than premature girls, similarly to what has been found in other studies [16]. The most important predictor of attention problems at age 18 months was mothers' educational level. Lower levels of maternal education seemed to predict more attention problems in the child. In fact, maternal education has been used as a marker of environmental risk in prematurity and as a proxy for quality of mother-child interactions and IQ [21].

Preliminary subgroup analyses with covariates were also carried out. Although these analyses were conducted in a reduced sample of children born very premature/with very low birth weight, there was a trend for an increased magnitude of the cross-lagged parameter from attention to language, pointing to a stronger precursor role of attention in severe prematurity. Further research is needed using samples of *very *premature and *very *low birth weight children. Some of the children in our "very premature" subgroup had actually birth weight above 1500 g.

In fact, our *PLBW *group can be regarded as having a relatively low medical risk since it was composed mostly of children born "mildly" premature and with relatively high birth weight. Furthermore, the environmental risk associated with mothers' demographic variables was also reduced. For example, the sample included a large percentage of women with higher education. Although this reflects the educational level in Norway for women in this age range (approximately 50% have higher education, according to Statistics Norway 2009), there was a slight overrepresentation of highly educated women in this sample (60%). With regards to representativeness of the overall MoBa sample, there is underrepresentation of women under 25 years, those living alone, mothers with more than two previous births and with previous stillbirths. Smokers are also underrepresented in the cohort [42]. Reduction of these unfavourable environmental factors might have impacted on the results by further decreasing the risk associated with prematurity in the current sample. It is therefore noteworthy that even in a relatively low-risk sample of *PLBW *children, we still found a precursor role of attention in relation to language. This effect might be greater in samples with higher levels of biological (e.g., *VLBW *children) or environmental (e.g., mother's low education) risk.

Some limitations of the study should be pointed out. The mother-report nature of the items used demands caution when comparing these findings with those obtained in observational studies. However, it has been shown that parents can offer accurate reports and constitute a valuable source of information [43,44]. Parents are good at reporting behaviours relevant to the developmental assessment of their children, especially those behaviours that can be observed and do not involve recall of past events. Parent report is problematic when parents have cognitive difficulties or low educational level [44,45]. The items used in our latent variables included assessment of present observable behaviours and our sample was composed of a large percentage of highly educated mothers. Furthermore, the parent-report items used in this study were drawn from well validated instruments. The CBCL is widely used and is considered to have good psychometric properties. In particular, the attention problem items of the CBCL have been able to distinguish referred and non-referred children [31]. There is also an extensive body of literature supporting the reliability and validity of language measures based on parental report [46-49], including studies using the ASQ. Its validity has been extensively demonstrated in samples including both normative and medical risk children (e.g., premature), with high overall agreement between the questionnaires and standardized assessments (88%) [45]. The validity of the ASQ communication scale has also been addressed. This scale is able to identify late language emergence, when using the scale as a whole but also at the single-item level [50]. Furthermore, the validity of the ASQ has been investigated in Norwegian samples. Decreased scores found in premature children have been regarded as supporting the construct validity of the Norwegian version of the scales [51]. Further studies are needed to assess the validity of the specific language and attention items included in the MoBa questionnaires, namely with regards to concurrent validity in relation to standardized assessments.

Another limitation concerns the items used in our language latent variables, which were drawn from a measure designed to be used as a screening instrument of language difficulties. Although we focused mainly on a *vulnerable *group of *PLBW *children and looked for correlates of attention *problems*, the language items included (especially at 36 months) might have constrained the variability of language skills observed, especially for the control group (ceiling effect). However, the very large size of the control group resulted in a reasonable spread of answers across all category responses in most analyses. Nevertheless, a more accurate way of framing our results would be to interpret them as pertaining to relations between attention problems and presence/absence of language delay. In fact, the ASQ high negative predictive value has supported its use as a screening tool in premature children [51].

Another issue concerning item selection must be mentioned. One of the indicators included in the latent variable measuring attention at 18 months is described in the CBCL manual as an overlapping item, present in both attention problems and hyperactivity disorder (see At18, item 3, in Appendix 1). One can argue that, conceptually, this indicator does not reflect "pure" attention problems. The item was nevertheless retained for reasons associated with viability of model building. Subsequent factor analyses revealed strong factor loadings for this item (over .70). In fact, this item was found to be one of the best to discriminate between moderate/severe symptoms and mild symptoms in a sample of clinically referred children, although these were older than the children participating in the current study [52].

Another potential limitation was the failure to account for multiple births. Some studies have found decreased language skills in twins when compared to singletons [53]. However, some basic comparative analyses including the premature low-birth-weight twins in our study revealed no disadvantage in terms of language and attention in relation to their singleton counterparts. Finally, variables such as child temperament, maternal sensitivity, and heritability of language and attention disorders should be controlled for in future studies, since they have been shown to relate to attention and language outcomes [54-56]. Items covering history of language delay and child temperament have been already incorporated in the MoBa questionnaires and can be used in future studies. Assessing maternal sensitivity presents more challenges due to very large sample sizes and use of self-report format in MoBa. We used mother's education as a proxy for family risk. Mother's education has been found to be importantly related to parental practices and home environment [57,58] and regarded as one of the best indicators of parenting behaviour [59]. Future studies should also concentrate on other subgroups of infants besides those born premature and with low birth weight, especially those born "small for gestational age" as the result of intrauterine growth restriction (low birth weight regardless of premature status) [60].

## Conclusions

This study represented a preliminary attempt to shed light on relations between attention problems and language ability in preterm low-birth-weight children. We found some preliminary evidence of a precursor role of attention problems in relation to language ability in prematurity. It is hoped that this research paves the way for future studies that can advance our understanding of the developmental pathways of attentional and linguistic skills over time and lead to better management of unfavourable outcomes associated with co-morbid language and attention difficulties. This can facilitate clarification of diagnoses such as learning disabilities, specific language impairment, and ADHD, leading to better treatment interventions and improved prognosis for the affected children.

## Competing interests

The authors declare that they have no competing interests.

## Authors' contributions

LAR was responsible for literature review, measure selection, study design, statistical analyses, and manuscript preparation. HDZ contributed to the study design, data preparation, and revision of the manuscript. SS contributed to establishing the measures of child development in the MoBa study, in particular the language ability measures, and participated in a discussion about measure selection. HA and NRB participated in conceptual discussions about measure selection, particularly the measures of attention problems. PM was responsible for conducting the cohort study and has participated in the critical revision and approval of the final manuscript. All authors read and approved the final manuscript.

## Pre-publication history

The pre-publication history for this paper can be accessed here:

http://www.biomedcentral.com/1471-2431/11/59/prepub

## Supplementary Material

Additional file 1 **Appendix 1**. Indicators used in the latent variables L18, L36, At18, and At36. Click here for file
