# Supplementary material for: Attention problems and language development in preterm low-birth-weight children: Cross-lagged relations from 18 to 36 months
Source: BMC Pediatr. 2011 Jun 29;11:59. doi: 10.1186/1471-2431-11-59 (PMC3163534; doi:10.1186/1471-2431-11-59)
Supplement: Additional file 1 — Appendix 1. Indicators used in the latent variables L18, L36, At18, and At36. [file 1471-2431-11-59-S1.DOC]

Appendix 1

L18

1. When you ask him/her does your child go into another room to find a familiar toy or object? (When you ask for instance: “where’s your ball?”, “go get your coat” or “go and get your blanket.”)
2. Does your child say eight or more words, in addition to “mama” and “dada”?
3. Without showing him/her first, does your child point to the correct picture when you say “show me the cat?” or “where is the dog?”

At18

1. Can’t concentrate, can’t pay attention for long.
2. Quickly shifts from one activity to another.
3. Can’t sit still, restless or hyperactive.

L36

1. Without showing him first, does your child point to the correct picture when you say, “where is the cat” or “where is the dog?” Your child must only point to the correct picture.
2. When you ask your child to point at his/her eyes, nose, hair, feet, ears, etc., does he/she point correctly at least to seven parts of the body?
3. Does your child use sentences made up of three or four words?
4. Without giving him/her help by pointing or using gestures, ask your child to “put the shoe on the table” and “put the book under the chair”. Does your child carry out both these directions correctly?
5. When looking at a picture book, does your child tell you what is happening or what action is taking place in the picture? You may ask, “what is the dog (or boy) doing”?
6. Can your child tell you at least two things about an object he/she is familiar with? If you say, for example “tell me about your ball”, will your child answer by saying “it is round, I can throw it, it is big”?

At36

1. Can’t concentrate, can’t pay attention for long.
2. Quickly shifts from one activity to another.
3. Becomes diverted or distracted by outside stimuli (sounds or events).
4. Often has difficulty sustaining attention in tasks or play activities.
5. Doesn’t seem to listen when he/she is being spoken to.
